# On implementation of a semi-analytic strategy to develop an analytical solution of a steady-state isothermal tube drawing model

**DOI:** 10.1038/s41598-022-11708-5

**Published:** 2022-05-10

**Authors:** Azhar Iqbal Kashif Butt, Nehad Ali Shah, Waheed Ahmad, Thongchai Botmart, Naeed Ahmad

**Affiliations:** 1 Department of Mathematics, Government College University, Lahore, 54000 Pakistan; 2 Department of Mechanical Engineering, Sejong University, Seoul, 05006 South Korea; 3 Department of Mathematics, Faculty of Science, Khon Kaen University, Khon Kaen, 40002 Thailand; 4 Department of Mathematics, Government Murray College, Sialkot, Pakistan

**Keywords:** Aerospace engineering, Mechanical engineering

## Abstract

In this paper, we consider an isothermal glass tube drawing model consisting of three coupled nonlinear partial differential equations. The steady-state solution of this model is required in order to investigate its stability. With the given initial and boundary conditions, it is not possible to determine an analytical solution of this model. The difficulty lies in determining the constants of integrations while solving the second order ordinary differential equation analytically appearing in the steady-state model. To overcome this difficulty, we present a numerical based approach for the first time to develop an analytical solution of the steady-state isothermal tube drawing model. We use a numerical technique called shooting method to convert the boundary value problem into a set of initial value problems. Once the model has been converted into a system of differential equations with initial values, an integrating technique is implemented to develop the analytical solution. The computed analytical solution is then compared with the numerical solution to better understand the accuracy of obtained solution with necessary discussions.

## Introduction

In glass industry, tubes are drawn through various manufacturing processes used to achieve the continuous production of glass tubes having correct wall thickness and diameter. The most commonly used are the Danner process and the Vello process^1–5^ having great importance in glass fabricating industry and are still in use today. In Danner process, glass is melted in a furnace to the stage where it is soft and pliable. Molten glass is then let to fall with low feeding speed $v_{0}$ on the surface of a cylindrical device called mandrel kept in a temperature controlled tank called oven. Mandrel is slightly inclined and hollow such that the air can be blown through it. By continuous rotation of mandrel about its axis of symmetry, the molten glass falling downward creates a smooth layer around the mandrel. It cools down gradually and takes the shape of a thick-walled hollow glass tube with all desired properties at just below the end of mandrel. The length of hot-forming zone is taken as *L*. It is then pulled out by a drawing machine with a drawing speed $v_{L}> v_{0}$. This ratio $v_{L}/v_{0}>1$ is called the draw ratio. Keeping a constant temperature in the hot-forming zone leads to develop an isothermal tube drawing model. The drawn tube is then conveyed straight by rollers to further process of cutting, finishing, polishing and packaging at the end of the spinline. This manufacturing process is explained and illustrated in^3^. For the Vello process, we refer to^1–5^.

**Figure 1:**
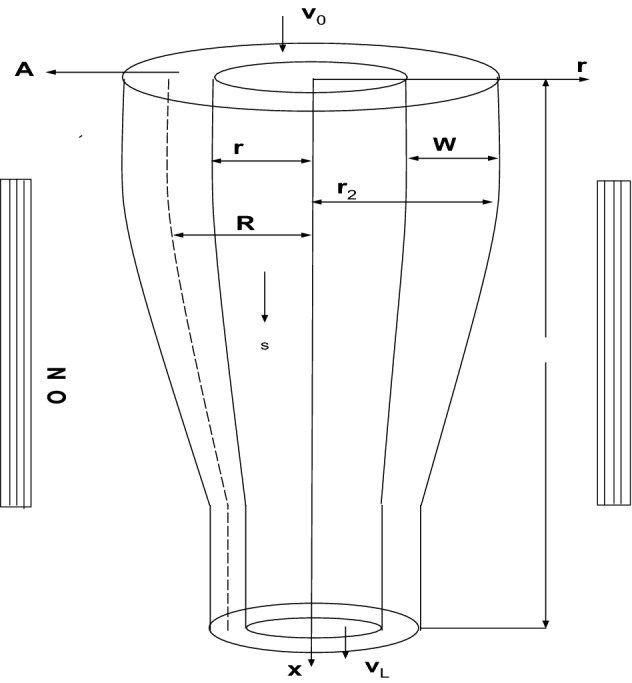
Diagram of a glass tube during production process.

The geometry of the drawn tube is illustrated in Fig. 1. The length of hot forming zone is denoted by *L*. Inner and outer radii, inside pressure, feeding and take up speeds, axis of symmetry of the tube during the production process are shown in Figure. All other parameters involved along with their numerical values are illustrated in Table 1.

**Table 1:** Summary of parametric values appearing in the isothermal model (5).

Parameter	Symbol	Approximate value	Units
Feeding speed	$v_{0}$	1	mm/s
Drawing speed	$v_{L}$	12	mm/s
Length of the hot-forming zone	*L*	1	m
Input viscosity	$\mu _{0}$	$5 \times 10^{5}$	Pa s
Inside pressure	$p_{s}$	420	Pa
Density	$\rho$	2500	${\text {kg}}/{\text {m}}^{3}$
Mean radius of the glass tube	$R_{0}$	30	mm
Initial area of the tube	$A_{0}$	1885	mm

The shaping parameters such as the wall thickness and cross-sectional area (or diameter) are the main characterizations of the drawn tube. In either of the manufacturing processes, the required shape of the tube can be maintained by the stream of the air gently blown through the mandrel. Insufficient quantity of the air blown through the mandrel can disturb the desired shape of the glass tube. As a result, this insufficient balance collapse the walls of the glass tube. Moreover, the geometry of the glass tube can be controlled by the parameters involved herein such as the glass temperature, the composition of the raw material, the pressure of the blowing air in the mandrel and the rate of draw. We have added a Fig. 1 to better understand the geometry of the drawn glass tube.

A variety of mathematical models both for the isothermal and non-isothermal tube drawing processes, with different levels of descriptions and needs, are available in the literature e.g., see^1–4^ and^6–11^. In^1–3,7^ numerical solutions of both the isothermal and non-isothermal models have been found and used to optimally control the geometry of the glass tube. In recent years, a variety of mathematical models representing physical and real world problems have been investigated for numerical solutions and stability analysis (for example see^12–24^). Thus, the concept of finding numerically accurate and exact solutions of real world problems has attracted attention from all over the world.

In this paper, we have considered an isothermal glass tube drawing model consisting of three coupled nonlinear partial differential equations of first and second order. The steady-state numerical solution of this model is required in order to investigate its stability. Recently, we have analyzed the stability of an isothermal tube drawing model and performed a complete mathematical analysis in^3^ by incorporating a steady state numerical solution. To better understand the physical model and to examine the accuracy of the obtained numerical solution, our objective here is to develop an analytical solution of the steady-state isothermal model. To start with, it is not possible to determine an analytical solution of this type of model with the given initial and boundary conditions. When we solve the second order ordinary differential equation appearing in the steady-state model, the difficulty lies in determining the constants of integrations. To overcome this concern, we utilize a numerical technique called shooting method to convert the boundary value problem into a set of initial value problems. An integrating technique is implemented to develop an analytical solution of the converted initial value problems. Similar approach has been suggested in^10,22^ to construct an analytical solution of a steady-state melt-spinning model. The semi-analytical approach developed in this paper has an advantage over other numerical methods that it may be applied directly to both linear and nonlinear systems of equations without the need for discretization, linearization, or perturbation.

The organization of the paper is as follows: In “Governing equations” section, we give a brief description of the mathematical model of an isothermal tube drawing process. A strategy converting a boundary value problem into a set of initial value problems is described in “Numerical approach” section. Analytical solution is developed in “Analytical solution” section and compared with the numerical solution in “Accuracy comparison” section. Conclusion is given in “Conclusions” section. We have included some leftover parts of the modeling of tube drawing process in the “Appendix”. In this section, we have included one example concerning the application of the analytical solution obtained through the implemented semi-analytic technique.

## Governing equations

In the literature, different types of models for the drawing processes with different level of demands and descriptions are available. A considerable amount of work has been carried out by different researchers^1–4,6,8,9^ and^11,25–28^ to model the tube drawing process. In this section, we briefly explain the mathematical model for an isothermal tube drawing process.

To model the tube drawing process, we consider an incompressible Newtonian flow of a molten glass between two free surfaces $r=r_{1}(z,t)$ and $r=r_{2}(z,t)$ where $r_{1}(z,t)$ and $r_{2}(z,t)$ respectively denote the inner and outer radii of the glass tube, and assume that temperature remains constant throughout the forming zone. Glass tube during production process is illustrated in Fig. 1. In the draw-down zone, the surface tension force and the inertial force acting upon the molten glass are insignificant and hence can be neglected. This kind of flow is governed by the equations

1a $$\begin{aligned}&\frac{\partial u}{\partial z}+\dfrac{1}{r}\frac{\partial }{\partial r}(rv)=0, \end{aligned}$$

1b $$\begin{aligned}&\frac{\partial p}{\partial r}=\frac{\partial }{\partial z}\left( \mu \frac{\partial v}{\partial z}\right) +\mu \left( \frac{\partial ^{2}v}{\partial r^{2}}+\dfrac{1}{r}\frac{\partial v}{\partial r}-\frac{v}{r^{2}}\right) +2\frac{\partial \mu }{\partial r}\frac{\partial v}{\partial r}+\frac{\partial \mu }{\partial z}\frac{\partial u}{\partial r}, \end{aligned}$$

1c $$\begin{aligned}&\frac{\partial p}{\partial z}=\frac{1}{r}\frac{\partial }{\partial r}\left( \mu r\frac{\partial v}{\partial z}\right) +\frac{1}{r}\frac{\partial }{\partial r}\left( \mu r\frac{\partial u}{\partial r}\right) +\rho g+\frac{\partial }{\partial z}\left( 2\mu \frac{\partial u}{\partial z}\right) . \end{aligned}$$

The Eqs. (1a)–(1c) are taken from^4^ and are known as the standard equations showing the axi-symmetric stokes flow. The first equation is the continuity equation and the last two equations are the momentum equations respectively in *r* and *z* directions. We denote the derivatives by subscripts *r* and *z* where *z* denotes the distance along the axis of the glass tube and *r* measures the distance perpendicular to it. The velocity of the molten glass is defined to be $\bar{\mathbf{v }}=(u,v)$ where *u* and *v* are the components of velocity $\bar{\mathbf{v }}$ along *z* and *r* direction respectively. The pressure, density and the acceleration due to gravity are denoted by *p*, $\rho$ and *g* respectively.

Now, at the free surfaces $r=r_{1}(z,t)$ and $r=r_{2}(z,t)$, it is necessary to specify the stress conditions and the kinematic conditions.

On the inner and outer surfaces of the glass tube, the stress conditions are given as:

1d $$\begin{aligned} \tau {\hat{n}}_{i}&=-p_{s}{\hat{n}}_{i} \quad on \; r=r_{1}, \end{aligned}$$

1e $$\begin{aligned} \tau {\hat{n}}_{o}&=0 \quad on \; r=r_{2}, \end{aligned}$$

where ${\hat{n}}_{i}$ and ${\hat{n}}_{o}$ are the unit normals on the surfaces $r=r_{1}$ and $r=r_{2}$ of the tube respectively defined as

$$\begin{aligned} {\hat{n}}_{i}=\dfrac{1}{\sqrt{1+\left( \dfrac{\partial r_{1}}{\partial z}\right) ^{2}}}\left( 1,-\dfrac{\partial r_{1}}{\partial z}\right) \quad \text {and} \quad {\hat{n}}_{o}=\dfrac{-1}{\sqrt{1+\left( \dfrac{\partial r_{2}}{\partial z}\right) ^{2}}}\left( 1,-\dfrac{\partial r_{2}}{\partial z}\right) . \end{aligned}$$

and $p_{s}$ is the inside pressure applied on the surface $r=r_{1}$ of the glass tube and $\tau$ is the stress tensor given as

$$\begin{aligned} \tau = \left( \begin{array}{cc} -p+2\mu \dfrac{\partial v}{\partial r} &{} \mu \left( \dfrac{\partial u}{\partial r}+\dfrac{\partial v}{\partial z}\right) \\ \mu \left( \dfrac{\partial u}{\partial r}+\dfrac{\partial v}{\partial z}\right) &{} -p+2\mu \dfrac{\partial u}{\partial z} \\ \end{array} \right) . \end{aligned}$$

The kinematic conditions are described as

1f $$\begin{aligned} v&=\frac{\partial r_{1}}{\partial t}+\frac{\partial r_{1}}{\partial z}u \quad on \; r=r_{1}, \end{aligned}$$

1g $$\begin{aligned} v&=\frac{\partial r_{2}}{\partial t}+\frac{\partial r_{2}}{\partial z}u \quad on \; r=r_{2}, \end{aligned}$$

and thus the stress conditions (1d)–(1e) can be expanded to give

1h $$\begin{aligned}&-\mu \left( \frac{\partial v}{\partial z}+\frac{\partial u}{\partial r}\right) \frac{\partial r_{1}}{\partial z}+\left( -p+2\mu \frac{\partial v}{\partial r}\right) =-p_{s} \quad on \; r=r_{1}, \end{aligned}$$

1i $$\begin{aligned}&-\left( -p+2\mu \frac{\partial u}{\partial z}\right) \frac{\partial r_{1}}{\partial z}+\mu \left( \frac{\partial u}{\partial r}+\frac{\partial v}{\partial z}\right) =p_{s}\frac{\partial r_{1}}{\partial z} \quad on \; r=r_{1}, \end{aligned}$$

1j $$\begin{aligned}&\mu \left( \frac{\partial v}{\partial z}+\frac{\partial u}{\partial r}\right) \frac{\partial r_{2}}{\partial z}=-p+2\mu \frac{\partial v}{\partial r}\quad on \; r=r_{2}, \end{aligned}$$

1k $$\begin{aligned}&\left( -p+2\mu \frac{\partial u}{\partial z}\right) \frac{\partial r_{2}}{\partial z}=\mu \left( \frac{\partial u}{\partial r}+\frac{\partial v}{\partial z}\right) \quad on \; r=r_{2}. \end{aligned}$$

where $\mu$ denotes the viscosity of the molten glass which remains constant at the given temperature.

To take benefit of the small parameters present in the problem, it is now convenient to convert the Eq. (1) into dimensionless form. For the dimensional quantities, the appropriate scaling is defined as:

$$\begin{aligned} z&=L{\tilde{z}},\; r=\varepsilon L{\tilde{r}},\; r_{1}=\varepsilon L{\tilde{r}}_{1},\; r_{2}=\varepsilon L{\tilde{r}}_{2},\; \mu =\mu _{0}{\tilde{\mu }},\\ u&=U{\tilde{u}},\; v=\varepsilon U{\tilde{v}},\; t=\frac{L}{U}{\tilde{t}},\; p=\frac{\mu _{0}U}{L}{\tilde{p}},\; p_{s}=\frac{\mu _{0}U}{L}\tilde{p_{s}}, \end{aligned}$$

where $\varepsilon =\dfrac{W}{L}\ll 1$. *W* is the width of the glass tube that has a very small value than the typical length of hot-forming zone *L*, *U* is the typical drawing speed and $\mu _{0}$ is the typical melt glass viscosity which has a constant value for the isothermal case. After dropping the bar notation, the system of governing equations in dimensionless form is given as follows:

2a $$\begin{aligned}&0=\dfrac{1}{r}\frac{\partial }{\partial r}(rv)+\frac{\partial u}{\partial z}, \end{aligned}$$

2b $$\begin{aligned}&\frac{\partial p}{\partial r}=\mu \left( \frac{\partial ^{2}v}{\partial r^{2}}+\frac{1}{r}\frac{\partial v}{\partial r}-\frac{v}{r^{2}}\right) +\varepsilon ^{2}\frac{\partial }{\partial z}\left( \mu \frac{\partial v}{\partial z}\right) +\frac{\partial \mu }{\partial z}\frac{\partial u}{\partial r}+2\frac{\partial \mu }{\partial r}\frac{\partial v}{\partial r}, \end{aligned}$$

2c $$\begin{aligned}&\varepsilon ^{2}\frac{\partial p}{\partial z}=\frac{1}{r}\frac{\partial }{\partial r}\left( \mu r\frac{\partial u}{\partial r}\right) +\varepsilon ^{2}\frac{\partial }{\partial z}\left( 2\mu \frac{\partial u}{\partial z}\right) +\varepsilon ^{2}\frac{1}{r}\frac{\partial }{\partial r}\left( \mu r\frac{\partial v}{\partial z}\right) +\varepsilon ^{2}{St}. \end{aligned}$$

where $St=\dfrac{\rho gL^{2}}{\mu _{0}U}$ is known as Stokes number.

The stress conditions (1h)–(1k) read as

2d $$\begin{aligned}&\left( -p+2\mu \frac{\partial v}{\partial r}\right) -\mu \left( \varepsilon ^{2}\frac{\partial v}{\partial z}+\frac{\partial u}{\partial r}\right) \frac{\partial r_{1}}{\partial z}=-p_{s} \quad on \; r=r_{1}, \end{aligned}$$

2e $$\begin{aligned}&\mu \left( \frac{\partial u}{\partial r}+\varepsilon ^{2}\frac{\partial v}{\partial z}\right) -\varepsilon ^{2}\left( -p+2\mu \frac{\partial u}{\partial z}\right) \frac{\partial r_{1}}{\partial z}=\varepsilon ^{2}p_{s}\frac{\partial r_{1}}{\partial z} \quad on \; r=r_{1}, \end{aligned}$$

2f $$\begin{aligned}&-p+2\mu \frac{\partial v}{\partial r}=\mu \left( \varepsilon ^{2}\frac{\partial v}{\partial z}+\frac{\partial u}{\partial r}\right) \frac{\partial r_{2}}{\partial z}\quad on \; r=r_{2}, \end{aligned}$$

2g $$\begin{aligned}&\mu \left( \frac{\partial u}{\partial r}+\varepsilon ^{2}\frac{\partial v}{\partial z}\right) =\varepsilon ^{2}\left( -p+2\mu \frac{\partial u}{\partial z}\right) \frac{\partial r_{2}}{\partial z} \quad on \; r=r_{1}. \end{aligned}$$

The kinematics conditions (1f)–(1g) become

2h $$\begin{aligned} v&=\frac{\partial r_{1}}{\partial t}+\frac{\partial r_{1}}{\partial z}u \quad on \; r=r_{1}, \end{aligned}$$

2i $$\begin{aligned} v&=\frac{\partial r_{2}}{\partial t}+\frac{\partial r_{2}}{\partial z}u \quad on \; r=r_{2}. \end{aligned}$$

The above model can be simplified by means of an asymptotic expansion, in which the inverse aspect ratio $\varepsilon$ is used as scaling parameter. Assuming the glass flow as a thin layer flow and ignoring the large aspect ratio of the flow, one derives the simplified equations to model the isothermal tube drawing process. In this derivation, the surface tension and inertial forces acting upon the molten glass have also been ignored due to their insignificant contributions. For details, we refer to^1–6^ and^9,27^. However, we have included the remaining considerations of the modeling process in “Appendix A” and reach at the following model equations representing the isothermal process:

3a $$\begin{aligned}&\frac{\partial A}{\partial t}+\frac{\partial }{\partial z}(vA)=0, \end{aligned}$$

3b $$\begin{aligned}&\frac{\partial }{\partial z}\left( 3\mu A\frac{\partial v}{\partial z}\right) +\rho gA=0, \end{aligned}$$

3c $$\begin{aligned}&\frac{\partial }{\partial t}(R^{2})+\frac{\partial }{\partial z}(vR^{2})=\frac{p_{s}}{16\pi \mu A}\left( 16\pi ^{2}R^{4}-A^{2}\right) , \end{aligned}$$

equipped with the initial conditions

3d $$\begin{aligned} A(z,0)=A_{0},\ R(z,0)=R_{0},\ \text {for}\ z\in {[0,L]}, \end{aligned}$$

and the boundary conditions

3e $$\begin{aligned} A(0,t)=A_{0},\ R(0,t)=R_{0},\ v(0,t)=v_{0},\ v(L,t)=v_{L},\ \text {for}\ t\ge 0, \end{aligned}$$

where $A_{0}$ and $R_{0}$ are the cross-sectional area and average radius of the glass tube at the time of entering the hot-forming zone, respectively. Acceleration due to gravity and density of the molten glass are denoted by *g* and $\rho$, respectively. Average radius *R* of the tube is defined as

$$\begin{aligned} R=\dfrac{1}{2}\left( r_1+r_2\right) . \end{aligned}$$

Equations in system (3) give us cross-sectional area *A*, velocity *v*, and average radius *R* of the glass tube. Width *W* of the tube can be determined by the equation

$$\begin{aligned} A=2\pi RW. \end{aligned}$$

Since the temperature throughout the forming zone of the tubing process remains constant, the viscosity $\mu$ of the melt glass also remains constant.

### Dimensionless form

We introduce the following dimensionless quantities

$$\begin{aligned} z^{*}=\dfrac{z}{L},\ t^{*}=\dfrac{v_{0}t}{L},\ A^{*}=\dfrac{A}{A_{0}},\ R^{*}=\dfrac{R}{R_{0}},\ v^{*}=\dfrac{v}{v_{0}},\ p^{*}=\dfrac{Lp_{s}}{\mu _{0}v_{0}},\ \mu ^{*}=\dfrac{\mu }{\mu _{0}}, \end{aligned}$$

into the model (3) to get the dimensionless equations

4a $$\begin{aligned}&\frac{\partial A}{\partial t}+\frac{\partial }{\partial z}(vA)=0, \end{aligned}$$

4b $$\begin{aligned}&\frac{\partial }{\partial z}(3A\frac{\partial v}{\partial z})+St A=0, \end{aligned}$$

4c $$\begin{aligned}&\frac{\partial }{\partial t}(R^{2})+\frac{\partial }{\partial z}(vR^{2})=\frac{\pi c p}{A}\left( R^{4}-\frac{A^{2}}{(4\pi c)^{2}}\right) , \end{aligned}$$

where we have removed the asterisk notation and

$$\begin{aligned} St=\frac{\rho gL^{2}}{\mu v_{0}}\quad \text {and}\quad c=\frac{R_{0}^{2}}{A_{0}}, \end{aligned}$$

are the dimensionless parameters.

The initial and boundary conditions are respectively transformed to

4d $$\begin{aligned}&A(z,0)=1,\ R(z,0)=1,\ \text {for all}\ z\in {[0,1]}, \end{aligned}$$

4e $$\begin{aligned}&A(0,t)=1,\ R(0,t)=1,\ v(0,t)=1,\ v(1,t)=v_{d},\ \text {for} \ t\ge 0, \end{aligned}$$

where $v_{d}=\dfrac{v_{L}}{v_{0}}>1$ is the draw ratio.

The steady state form of the model (4) is written as

5a $$\begin{aligned}&\dfrac{d}{dz}(vA)=0, \end{aligned}$$

5b $$\begin{aligned}&\dfrac{d}{dz}(3A\dfrac{dv}{dz})+StA=0, \end{aligned}$$

5c $$\begin{aligned}&\dfrac{d}{dz}(vR^{2})=\dfrac{\pi c p}{A}\left( R^{4}-\dfrac{A^{2}}{(4\pi c)^{2}}\right) , \end{aligned}$$

subject to the conditions:

5d $$\begin{aligned} A(0)=1,\ v(0)=1,\ v(1)=v_{d},\ R(0)=1. \end{aligned}$$

The system (5) is a system of coupled nonlinear ordinary differential equations. We are interested in developing an analytical solution of the nonlinear system (5). However, we have already proved the existence and uniqueness of the solution of steady state isothermal tube drawing model (5) in^3^. For details, we refer the reader to^3^.

## Numerical approach

There are different techniques available in the literature to solve boundary value problems analytically. The obtained general solutions contains arbitrary constant which can be determined by using boundary conditions. However, there exist some problems (e.g. problem (5) and see^10,22^) where it is not possible to find integration constants with the given conditions. It is interesting to mention that integration constants can be found if boundary value problem (BVP) is transformed to an initial value problem (IVP).

Shooting method is a technique that transforms a BVP of the form

$$\begin{aligned} \dfrac{d^2\phi }{dz^2}=f\Big (z, \phi , \dfrac{d\phi }{dz}\Big )\ \ \phi (a)=\alpha ,\ \phi (b)=\beta , \end{aligned}$$

into an IVP

6 $$\begin{aligned} \dfrac{d^2\phi }{dz^2}=f\Big (z, \phi , \dfrac{d\phi }{dz}\Big )\ \ \phi (a)=\alpha ,\ \dfrac{d\phi }{dz}(a)=s, \end{aligned}$$

where *s* is a guess to be determined in such a way that it shoots $\phi (b)$.

We apply RK4 method to the second order differential Eq. (6) to determine approximation $s_{i}, i = 1, 2, \ldots$ for $\phi (b)$. Let the first guess $s_{1}$ be taken as

$$\begin{aligned} s_{1}=\dfrac{d\phi }{dz}(a)\approx \dfrac{\phi (b)-\phi (a)}{b-a} \end{aligned}$$

RK4 method with $s_{1}$ gives us an approximation $\beta _{1}$ for $\phi (b)$. If the absolute error $|\beta _{1}-\phi (b)|$ is less than some pre-assigned tolerance, we stop, otherwise we refine our guess by considering

$$\begin{aligned} s_{2}=2s_{1}, \end{aligned}$$

and compute $\beta _{2}$ by RK4 method. If the error $|\beta _{2}-\phi (b)|$ agrees to pre-assigned tolerance, we stop. In the case of disagreement, we make further guesses using the secant formula

7 $$\begin{aligned} s_{i+1}=\dfrac{d\phi }{dz}(a)\approx s_{i}-e(s_{i})\Bigg [\dfrac{s_{i-1}-s_{i}}{e(s_{i-1})-e(s_{i})}\Bigg ],\ \ i=2,3,\ldots \end{aligned}$$

where

$$\begin{aligned} e(s_{j})=\beta _{j}-\phi (b),\ \ j=1,2,\ldots \end{aligned}$$

is the error of approximation. RK4 method is continued each time with a new guess $s_{i}, i = 3, 4, \ldots$ computed from (7) until $\beta _{i}$ agrees with the value $\phi (b)$ to pre-set tolerance.

Once we able to compute a most suitable guess $s_{i}\rightarrow s$, we are able to transform the BVP to IVP, and hence an analytical solution of the problem of type given in (6) can be found.

## Analytical solution

With the initial conditions (5d), the Eq. (5a) is solved to get

8 $$\begin{aligned} A(z)=\dfrac{1}{v(z)},\quad v(z)>0,\quad z\in {\Omega }=[0, 1]. \end{aligned}$$

Equation (5b) can now be put in the form

9a $$\begin{aligned} \dfrac{d^{2}v}{dz^{2}}-\dfrac{1}{v}\left( \dfrac{dv}{dz}\right) ^{2}=-\dfrac{1}{3}St, \end{aligned}$$

along with the conditions

9b $$\begin{aligned} v(0)=1,\ v(1)=v_{d}. \end{aligned}$$

With the usual techniques, it is not an easy task to develop an analytical solution of the BVP (9). We use a non-conventional approach to build an analytical solution of the given nonlinear model. We convert the BVP (9) into two IVPs. For this purpose, we set

10 $$\begin{aligned} \dfrac{dv}{dz}=w, \end{aligned}$$

and

$$\begin{aligned} \dfrac{d^{2}v}{dz^{2}}=w \dfrac{dw}{dv}. \end{aligned}$$

Then the IVPs corresponding to BVP (9) are defined as

11a $$\begin{aligned} \dfrac{dv}{dz}&=w, \end{aligned}$$

11b $$\begin{aligned} w\dfrac{dw}{dv}-\dfrac{1}{v}w^2&=-\dfrac{1}{3}St, \end{aligned}$$

along with the conditions

11c $$\begin{aligned} v(0)=1,\ w(0)=w_0. \end{aligned}$$

where $w_{0}$ is approximated value determined by shooting the value of *v* at 1 i.e., $v(1) = v_{d}$. (The process of finding the approximation $w_{0}=s$ (say) by shooting method is explained in “Numerical approach” section).

The solution of first order non-linear ordinary differential Eq. (11b) is determined to give

12 $$\begin{aligned} w=\sqrt{c_{1}v^{2}+\dfrac{2St}{3}v}, \end{aligned}$$

where $c_{1}$ is a constant of integration and is computed to give

13 $$\begin{aligned} c_{1}=(w_0)^{2}-\dfrac{2}{3}St, \end{aligned}$$

Solution (12) can be further re-written as

14 $$\begin{aligned} \dfrac{dv}{dz}=\sqrt{c_{1}v^{2}+\dfrac{2St}{3}v}. \end{aligned}$$

Separating the variables and then integrating, we reach at

15 $$\begin{aligned} \dfrac{1}{\sqrt{c_{1}}}\cosh ^{-1}(3c_{1}St^{-1}v+1)=z+c_{2}, \end{aligned}$$

where $c_{2}$ is another constant of integration.

Solving for *v*, we obtain

16 $$\begin{aligned} v=\dfrac{1}{3c_{1}}St\Bigg (-1+\cosh \Big (\sqrt{c_{1}}(z+c_{2})\Big )\Bigg ). \end{aligned}$$

Equation (15) in view of condition (5d) gives us the value of $c_{2}$, i.e.

17 $$\begin{aligned} c_{2}=\dfrac{1}{\sqrt{c_{1}}}\cosh ^{-1}\Big (3c_{1}St^{-1}+1\Big ). \end{aligned}$$

Equation (16) along with $c_{1}$ and $c_{2}$ respectively given in (13) and (17) represents an analytical solution of Eq. (5b).

Next we put the steady state solutions for *A* and *v* in Eq. (5c) to get

18 $$\begin{aligned} \dfrac{du}{dz}-(\pi c p)u^{2}+\left( \dfrac{\dfrac{St}{3\sqrt{c_{1}}}\sinh \sqrt{c_{1}}(z+c_{2})}{-\dfrac{St}{3c_{1}}+\dfrac{St}{3c_{1}}\cosh \sqrt{c_{1}}(z+c_{2})}\right) u =\dfrac{-\pi c p}{\Bigg (4\pi c\Big (-\dfrac{St}{3c_{1}}+\dfrac{St}{3 c_{1}}\cosh \sqrt{c_{1}}(z+c_{2})\Big )\Bigg )^{2}}, \end{aligned}$$

where $$u=R^{2}$$.

Equation (18) is a well-known Riccati’s equation^29^ whose solution is supposed to be of the form

19 $$\begin{aligned} u=u_{1}+\dfrac{1}{y}, \end{aligned}$$

where one solution $u_{1}$, guessed by hit and trial procedure, is given as

$$\begin{aligned} u_{1}=\dfrac{1}{4\pi c\left( -\dfrac{St}{3c_{1}}+\dfrac{St}{3c_{1}}\cosh \sqrt{c_{1}}(z+c_{2})\right) }. \end{aligned}$$

and *y* is any nonzero unknown function of *z*.

Differentiating (19) with respect to *z* and then putting the values of *u* and $\dfrac{du}{dz}$ in Eq. (18), we reach at

$$\begin{aligned} \dfrac{dy}{dz}+\left( \dfrac{\dfrac{p}{2}-\dfrac{St}{3\sqrt{c_{1}}}\sinh \sqrt{c_{1}}(z+c_{2})}{-\dfrac{St}{3c_{1}}+\dfrac{St}{3c_{1}}\cosh \sqrt{c_{1}}(z+c_{2})}\right) y=-\pi c p, \end{aligned}$$

which is a first order linear ordinary differential equation in y. After few steps of calculations, the solution of this differential equation is found to be

20 $$\begin{aligned} y=(-2\pi c)A+c_{3}B, \end{aligned}$$

where $c_{3}$ is a constant of integration, and *A*(*z*), *B*(*z*) are given as

$$\begin{aligned} A(z)&=-\frac{St}{3c_{1}}+\frac{St}{3c_{1}}\cosh \sqrt{c_{1}}(z+c_{2}),\\ B(z)&=\dfrac{-\dfrac{St}{3c_{1}}+\dfrac{St}{3c_{1}}\cosh (\sqrt{c_{1}}(z+c_{2}))}{exp\Bigg ({-\frac{3\sqrt{c_{1}}p}{2St}\Big (\coth (\sqrt{c_{1}}(z+c_{2}))+csch(\sqrt{c_{1}}(z+c_{2}))\Big )}\Bigg )}. \end{aligned}$$

Using (19) and the condition (5d), we get

21 $$\begin{aligned} c_{3}=(4\pi c)k_{1}+(2\pi c)k_{2}, \end{aligned}$$

where

$$\begin{aligned} k_{1}&=\dfrac{exp\Bigg (\dfrac{-3\sqrt{c_{1}}p}{2St}\Big (\coth \sqrt{c_{1}}c_{2}+csch\sqrt{c_{1}}c_{2}\Big )\Bigg )}{\Bigg (4\pi c\Big (-\dfrac{St}{3c_{1}}+\dfrac{St}{3c_{1}}\cosh \sqrt{c_{1}}c_{2}\Big )-1\Bigg )}, \end{aligned}$$

and

$$\begin{aligned} k_{2}&=exp\Bigg ({\frac{-3\sqrt{c_{1}}p}{2St}\Big (\coth \sqrt{c_{1}}c_{2}+csch\sqrt{c_{1}}c_{2}\Big )}\Bigg ). \end{aligned}$$

Substituting $c_{3}$ in (20), we obtain

$$\begin{aligned} \dfrac{1}{y}=\dfrac{E}{(-2\pi c)D(z)\times E+(4\pi c)D(z)\times e^{g(z)}+(2\pi c)D(z)\times Ee^{g(z)}}. \end{aligned}$$

Therefore,

$$\begin{aligned} u=\dfrac{1}{(4\pi c)D(z)}+\dfrac{E}{(-2\pi C)E\times D(z)+(4\pi c)D(z)\times e^{g(z)}+(2\pi c)E\times D(z)\times e^{g(z)}}, \end{aligned}$$

where

$$\begin{aligned} D(z)&=-\dfrac{St}{3c_{1}}+\dfrac{St}{3c_{1}}\cosh \Big (\sqrt{c_{1}}(z+c_{2})\Big ),\\ E&= 4\pi c\left( -\dfrac{St}{3c_{1}}+\dfrac{St}{3c_{1}}\cosh \sqrt{c_{1}}c_{2}\right) ,\\ g(z)&=\dfrac{3\sqrt{c_{1}}p}{2St}\Big (\coth \sqrt{c_{1}}(z+c_{2})+csch\sqrt{c_{1}}(z+c_{2})\Big )-\dfrac{3\sqrt{c_{1}}p}{2St}\Big (\coth \sqrt{c_{1}}c_{2}-csch\sqrt{c_{1}}c_{2}\Big ), \end{aligned}$$

and hence

22 $$\begin{aligned} R=\sqrt{u}, \end{aligned}$$

is an analytical solution of the Eq. (5c) along with $c_{1}$ and $c_{2}$ given respectively in (13) and (17). Thus, the solutions given in (8), (16) and (22) together constitute an analytical solution of the steady-state model (5).

## Accuracy comparison

**Figure 2:**
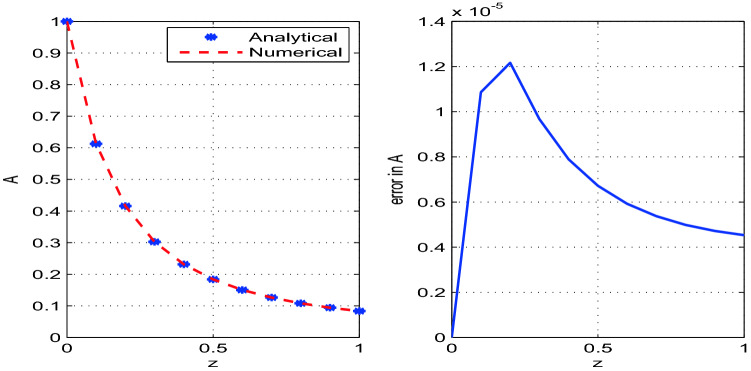
Analytical and numerical solutions for the steady state variable *A* and the corresponding errors between the solutions with discretization step size $h=0.1$.

**Figure 3:**
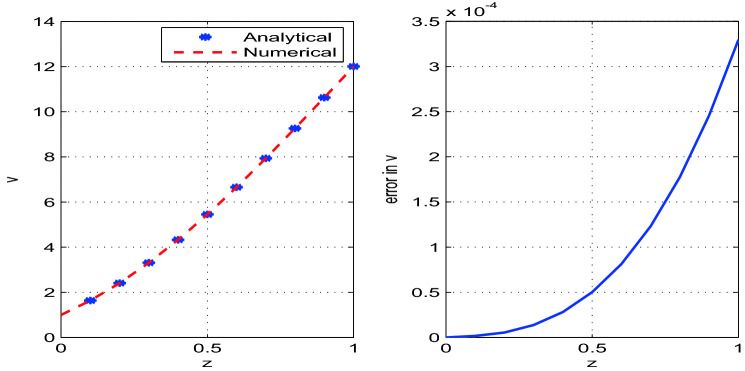
Analytical and numerical solutions for the steady state variable *v* and the corresponding errors between the solutions with discretization step size $h=0.1$.

**Figure 4:**
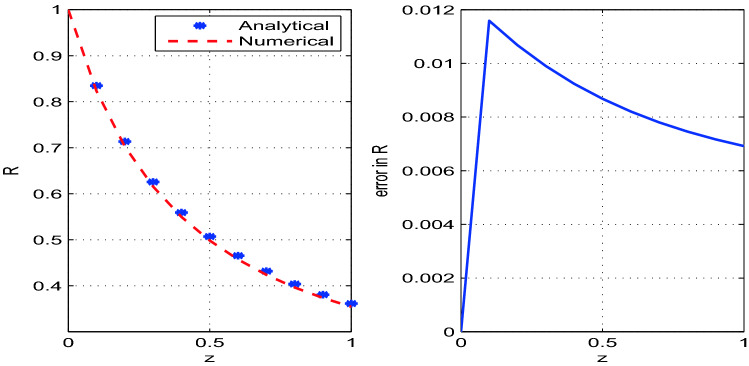
Analytical and numerical solutions for the steady state variable *R* and the corresponding errors between the solutions with discretization step size $h=0.1$.

**Figure 5:**
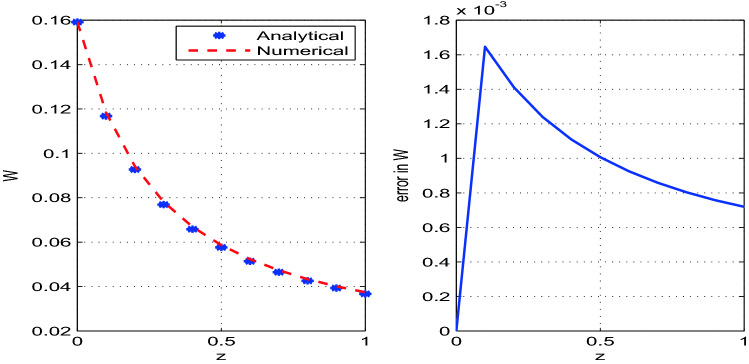
Analytical and numerical solutions for the steady state variable *W* and the corresponding errors between the solutions with discretization step size $h=0.1$.

The developed analytical solution is plotted and compared with the numerical solution in Figs. 2, 3, 4. The parametric values used in the model are given in Table 1. The solutions obtained by RK4 method are reliable and competitive with the analytical solutions. We observe that the errors between numerical and analytical solutions for the steady state variables *A*, *v* and *R* are negligibly small (see Table 2). We also determine *W*, the width of the glass tube, by using the relation $A=2\pi R W$. The steady state analytical and numerical solutions for *W* and the corresponding error are also shown in the Fig. 5. It is observed from Table 2 that error between the numerical and analytical solution for *A*, *v*, and *R* is maximum at $z=0.2$, $z=1.0$, and $z=0.1$ respectively. Moreover, the error for the solution *W* is maximum at $z=0.1$.

**Table 2:** Summary of errors in solutions for $z\in [0,1]$ using step size $h=0.1$.

*z*	Error in *A*	Error in *v*	Error in *R*	Error in *W*
0.0	0.00	0.00	0.00	0.00
0.1	$1.08\times 10^{-6}$	$1.70\times 10^{-7}$	$1.16\times 10^{-2}$	$1.60\times 10^{-6}$
0.2	$1.21\times 10^{-6}$	$5.40\times 10^{-7}$	$1.07 \times 10^{-2}$	$1.40\times 10^{-6}$
0.3	$9.67\times 10^{-7}$	$1.37\times 10^{-6}$	$9.90 \times 10^{-3}$	$1.20\times 10^{-6}$
0.4	$7.89\times 10^{-7}$	$2.82\times 10^{-6}$	$9.20 \times 10^{-3}$	$1.10\times 10^{-6}$
0.5	$6.72\times 10^{-7}$	$5.01\times 10^{-6}$	$8.70 \times 10^{-3}$	$1.00\times 10^{-6}$
0.6	$5.92\times 10^{-7}$	$8.12\times 10^{-6}$	$8.20 \times 10^{-3}$	$9.00\times 10^{-7}$
0.7	$5.37\times 10^{-7}$	$1.23\times 10^{-5}$	$7.80 \times 10^{-3}$	$9.00\times 10^{-7}$
0.8	$4.99\times 10^{-7}$	$1.77\times 10^{-5}$	$7.50 \times 10^{-3}$	$8.00\times 10^{-7}$
0.9	$4.72\times 10^{-7}$	$2.45\times 10^{-5}$	$7.20 \times 10^{-3}$	$8.00\times 10^{-7}$
1.0	$4.53\times 10^{-7}$	$3.29\times 10^{-5}$	$6.90 \times 10^{-3}$	$7.00\times 10^{-7}$

## Conclusions

In this work, we have presented a numerical based approach to find analytical solution of a boundary value problem where one cannot find constants of integration with the given boundary conditions. The approach is to convert a boundary value problem into initial value problems and then to develop an analytical solution of the resulting problems with the usual methods. To explain the approach, we have developed an analytical solution of a steady-state model of the isothermal tube drawing process with the help of shooting method. The obtained analytical solution is almost in agreement with the numerical solution that justifies our approach.

## Figures and Tables

**Figure 6:**
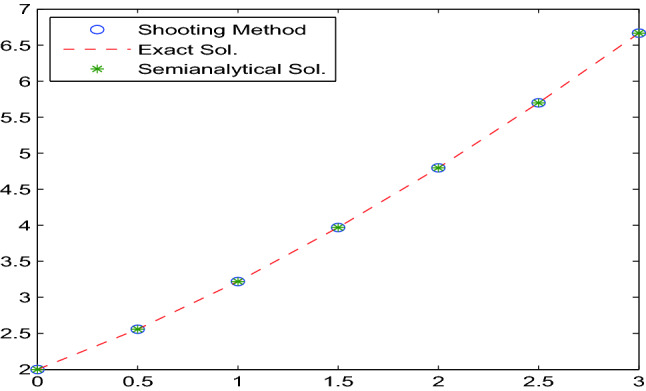
Comparison of exact solution and solutions obtained through the shooting method and developed semi-analytic technique using discretization step size $h=0.5$.

**Figure 7:**
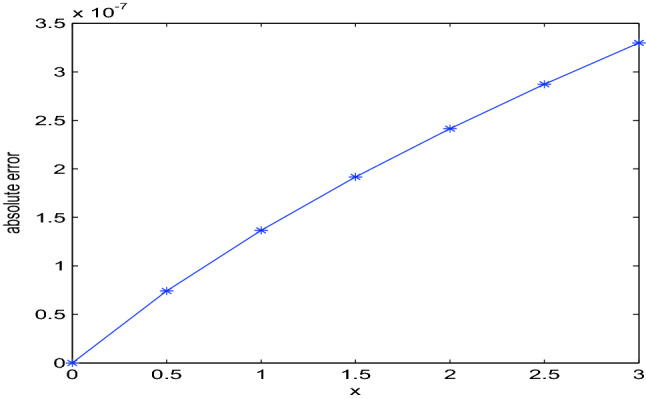
Error between the exact and semi-analytic solutions with discretization step size $h=0.5$.

**Figure 8:**
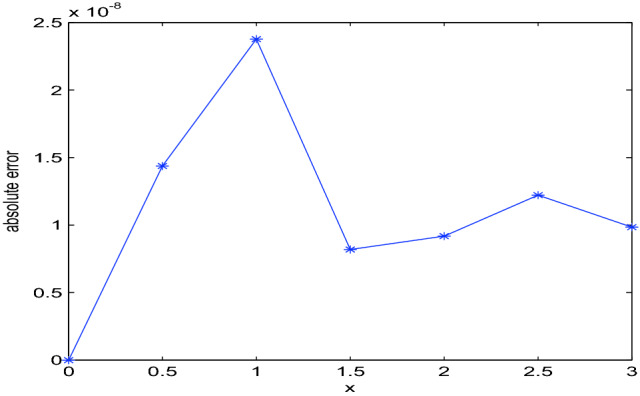
Error between the derived semi-analytic solution and that obtained through the shooting method using discretization step size $h=0.5$.

## Appendix

In this section, we present the remaining part of the modeling of tube drawing process. Moreover, one counter example concerning the application of the analytical solution derived through the proposed semi-analytic technique is demonstrated.

### Appendix A: Modeling tube drawing process

To simplify the system of Eq. (2), we take the advantage of the small parameter $\varepsilon$ present in the system (2) and exploit it. Note that the parameter $\varepsilon$ appears only in even powers of $\varepsilon$. Therefore, we use the following expansions of the dependent variables

$$\begin{aligned} u&=u_{0}+\varepsilon ^{2}u_{1}+{\mathscr {O}}(\varepsilon ^{4}),\\ v&=v_{0}+\varepsilon ^{2}v_{1}+{\mathscr {O}}(\varepsilon ^{4}),\\ p&=p_{0}+\varepsilon ^{2}p_{1}+{\mathscr {O}}(\varepsilon ^{4}).\\&\vdots \end{aligned}$$

We substitute the above expansions into the system of Eq. (2) and comparing the coefficients of like powers of $\varepsilon$ to get simplified model equations.

From the Eqs. (2a)–(2c), the leading-order contributions are respectively given as

23a $$\begin{aligned}&0=\dfrac{1}{r}\frac{\partial }{\partial r}(rv_{0})+\frac{\partial u_{0}}{\partial z}, \end{aligned}$$

23b $$\begin{aligned}&\frac{\partial p_{0}}{\partial r}=\mu \left( \frac{\partial ^{2}v_{0}}{\partial r^{2}}+\dfrac{1}{r}\frac{\partial v_{0}}{\partial r}-\frac{v_{0}}{r^{2}}\right) +\frac{\partial \mu }{\partial z}\frac{\partial u_{0}}{\partial r}+2\frac{\partial \mu }{\partial r}\frac{\partial v_{0}}{\partial r}, \end{aligned}$$

23c $$\begin{aligned}&0=\frac{\partial }{\partial r}\left( \mu r\frac{\partial u_{0}}{\partial r}\right) . \end{aligned}$$

The kinematic conditions (2h)–(2i) and the stress conditions (2d)–(2g) respectively give the following leading-order contributions.

23d $$\begin{aligned}&v_{0}=\frac{\partial r_{1}}{\partial t}+\frac{\partial r_{1}}{\partial z}u_{0} \quad \text {on} \; r=r_{1}, \end{aligned}$$

23e $$\begin{aligned}&v_{0}=\frac{\partial r_{2}}{\partial t}+\frac{\partial r_{2}}{\partial z}u_{0} \quad \text {on} \; r=r_{2}, \end{aligned}$$

23f $$\begin{aligned}&-p_{0}+2\mu \frac{\partial v_{0}}{\partial r} =-p_{s0} \quad \text {on} \; r=r_{1}, \end{aligned}$$

23g $$\begin{aligned}&\frac{\partial u_{0}}{\partial r}=0 \quad \text {on} \; r=r_{1}, \end{aligned}$$

23h $$\begin{aligned}&-p_{0}+2\mu \frac{\partial v_{0}}{\partial r}=0 \quad \text {on} \; r=r_{2}, \end{aligned}$$

23i $$\begin{aligned}&\frac{\partial u_{0}}{\partial r}=0 \quad \text {on} \; r=r_{2}. \end{aligned}$$

The leading-order momentum equation (23c) along with the leading-order conditions (23g), (23i) gives us

$$\begin{aligned} u_{0}=u_{0}(z,t), \end{aligned}$$

which shows that the leading-order axial velocity $u_{0}$ is not the function of *r*.

Thus the continuity equation (23a) reduces to

24 $$\begin{aligned} v_{0}=-\dfrac{r}{2}\dfrac{\partial u_{0}}{\partial z}+\dfrac{1}{r}G(z,t), \end{aligned}$$

where *G*(*z*, *t*) is to be found.

By the Eq. (24) and the boundary conditions (23f) and (23h), we obtain

25a $$\begin{aligned}&-p_{0}-\dfrac{2\mu }{r_{1}^{2}}G(z,t)-\mu \frac{\partial u_{0}}{\partial z} +p_{s0}=0, \end{aligned}$$

25b $$\begin{aligned}&-p_{0}-\dfrac{2\mu }{r_{2}^{2}}G(z,t)-\mu \frac{\partial u_{0}}{\partial z}=0. \end{aligned}$$

Solving the Eq. (25) for *G*(*z*, *t*) and $p_{0}$, we get

26 $$\begin{aligned}&G(z,t)=\dfrac{p_{s0}r_{1}^{2}r_{2}^{2}}{2\mu (r_{2}^{2}-r_{1}^{2})}, \end{aligned}$$

27 $$\begin{aligned}&p_{0}=-\dfrac{p_{s0}r_{1}^{2}}{r_{2}^{2}-r_{1}^{2}}-\mu \frac{\partial u_{0}}{\partial z}. \end{aligned}$$

By using the kinematic boundary condition (23d), the Eq. (24) at boundary $r=r_{1}$ is written as

28 $$\begin{aligned} -\dfrac{r_{1}}{2}\frac{\partial u_{0}}{\partial z}+\dfrac{1}{r_{1}}G(z,t)=\frac{\partial r_{1}}{\partial t}+\frac{\partial r_{1}}{\partial z}u_{0}, \end{aligned}$$

Now using (26), we obtain

29a $$\begin{aligned} \frac{\partial }{\partial t}(r_{1}^{2})+\dfrac{\partial }{\partial z}(u_{0}r_{1}^{2})=\dfrac{p_{s0}r_{1}^{2}r_{2}^{2}}{\mu (r_{2}^{2}-r_{1}^{2})}. \end{aligned}$$

Similarly the Eq. (24) at boundary $r=r_{1}$ gives us

29b $$\begin{aligned} \frac{\partial }{\partial t}(r_{2}^{2})+\dfrac{\partial }{\partial z}(u_{0}r_{2}^{2})=\dfrac{p_{s0}r_{1}^{2}r_{2}^{2}}{\mu (r_{2}^{2}-r_{1}^{2})}. \end{aligned}$$

Equation (29a) gives us the inner radius $r_{1}$ and the Eq. (29b) gives the outer radius $r_{2}$ of the glass tube. We do some manipulations with the Eq. (29) to obtain a differential equation for mean radius *R* in a dimensional form that is,

30 $$\begin{aligned} \frac{\partial }{\partial t}(R^{2})+\dfrac{\partial }{\partial z}(uR^{2})=\frac{p_{s}}{16\pi \mu A}(16\pi ^{2}R^{4}-A^{2}), \end{aligned}$$

where *R* in terms of $r_{1}$ and $r_{2}$ is the mean radius of the glass tube and is given by the relation

$$\begin{aligned} R=\frac{r_{1}+r_{2}}{2}, \end{aligned}$$

and $A=2\pi rW$ is the cross-sectional area of the glass tube where *W* is the width of the glass tube defined by the relation

31 $$\begin{aligned} W=r_{2}-r_{1}. \end{aligned}$$

Equations (29) yield us the continuity equation

32 $$\begin{aligned} \dfrac{\partial }{\partial t}(r_{2}^{2}-r_{1}^{2})+\dfrac{\partial }{\partial z}(u_{0}(r_{2}^{2}-r_{1}^{2}))=0. \end{aligned}$$

In dimensional form, the Eq. (32) becomes as

33 $$\begin{aligned} \frac{\partial A}{\partial t}+\frac{\partial }{\partial z}(uA)=0. \end{aligned}$$

Since $u_{0}$ and $\mu$ are not depending or *r*. Therefore, the leading-order *r*-momentum equation (23b) yields

$$\begin{aligned} \frac{\partial p_{0}}{\partial r}=0,\ \Rightarrow p_{0}=p_{0}(z,t), \end{aligned}$$

which mean that the leading-order pressure is not a function of *r*.

We consider the *z*-momentum equation to get equations in closed form for the tube drawing process.

34 $$\begin{aligned} \frac{\partial p_{0}}{\partial z}=\dfrac{\mu }{r}\frac{\partial }{\partial r}\left( r\frac{\partial u_{1}}{\partial r}\right) +\frac{\partial }{\partial z}\left( 2\mu \frac{\partial u_{0}}{\partial z}\right) +\dfrac{\mu }{r}\frac{\partial }{\partial r}\left( r\frac{\partial v_{0}}{\partial z}\right) +St. \end{aligned}$$

Multiplying the Eq. (34) by *r* and then integrating from $r=r_{1}$ to $r=r_{2}$, we get

35 $$\begin{aligned} \dfrac{r_{2}^{2}-r_{1}^{2}}{2}\left( \frac{\partial p_{0}}{\partial z}-\frac{\partial }{\partial z}\left( 2\mu \frac{\partial u_{0}}{\partial z}\right) +\mu \frac{\partial ^{2}u_{0}}{\partial z^{2}}-St\right) =\mu \left( r\dfrac{\partial u_{1}}{\partial r}\Big |_{r=r_{2}}-r\dfrac{\partial u_{1}}{\partial r}\Big |_{r=r_{1}}\right) . \end{aligned}$$

The boundary conditions of order $\varepsilon ^{2}$ for the normal stress are

36a $$\begin{aligned}&\mu \left( \frac{\partial u_{1}}{\partial r}+\frac{\partial v_{0}}{\partial z}\right) -\left( -p_{0}+2\mu \frac{\partial u_{0}}{\partial z}\right) \frac{\partial r_{1}}{\partial z}=p_{s0}\frac{\partial r_{1}}{\partial z} \quad on \; r=r_{1}, \end{aligned}$$

36b $$\begin{aligned}&\mu \left( \frac{\partial u_{1}}{\partial r}+\frac{\partial v_{0}}{\partial z}\right) =\left( -p_{0}+2\mu \frac{\partial u_{0}}{\partial z}\right) \frac{\partial r_{2}}{\partial z} \quad on \; r=r_{2}. \end{aligned}$$

Differentiating Eq. (24) with respect to *z* and then substituting the value of $\dfrac{\partial v_{0}}{\partial z}$ in the stress condition (36), we get

37a $$\begin{aligned}&\mu \frac{\partial u_{1}}{\partial r}|_{r=r_{1}}=\dfrac{\mu r_{1}}{2}\dfrac{\partial ^{2}u_{0}}{\partial z^{2}}-\dfrac{1}{r_{1}}\mu \frac{\partial G}{\partial z}+\left( -p_{0}+2\mu \frac{\partial u_{0}}{\partial z}\right) \frac{\partial r_{1}}{\partial z}+p_{s0}\frac{\partial r_{1}}{\partial z}, \end{aligned}$$

37b $$\begin{aligned}&\mu \frac{\partial u_{1}}{\partial r}|_{r=r_{2}}=\dfrac{\mu r_{2}}{2}\dfrac{\partial ^{2}u_{0}}{\partial z^{2}}-\dfrac{1}{r_{2}}\mu \frac{\partial G}{\partial z}+\left( -p_{0}+2\mu \frac{\partial u_{0}}{\partial z}\right) \frac{\partial r_{2}}{\partial z}. \end{aligned}$$

Differentiating $p_{0}$ with respect to *z* and using the conditions (37), the momentum equation (35), after certain simplification, becomes

$$\begin{aligned} \frac{\partial }{\partial z}\left( 3\mu (r_{2}^{2}-r_{1}^{2})\frac{\partial u_{0}}{\partial z}\right) +(r_{2}^{2}-r_{1}^{2})St=0, \end{aligned}$$

or in a dimensional form

38 $$\begin{aligned} \frac{\partial }{\partial z}\left( 3\mu A\frac{\partial u}{\partial z}\right) +\rho gA=0. \end{aligned}$$

For simplicity, we dropped the zero subscripts of the leading order quantities present in the derived Eqs. (30), (33) and (38). Moreover, we need to attach these derived equations with the initial and boundary conditions.

The initial conditions read as:

39a $$\begin{aligned} A(z,t=0)=A_{0},\ r(z,t=0)=r_{0}, \ \text {for all}\ z\in [0,L]. \end{aligned}$$

The boundary conditions are:

39b $$\begin{aligned} A(z=0,t)=A_{0},\ r(z=0,t)=r_{0},\ u(z=0,t)=u_{0},\ u(z=L,t)=u_{L},\ \forall \ t\ge 0, \end{aligned}$$

The derived model Eqs. (30), (33), (38) along with the initial conditions (39a) and the boundary conditions (39b) are highly nonlinear, coupled and describe an isothermal tube drawing process. To make it more readable, we replace *u* by *v*. Then the model for the isothermal tube drawing process in a dimensional form is given as:

40a $$\begin{aligned}&\frac{\partial A}{\partial t}+\frac{\partial }{\partial z}(vA)=0, \end{aligned}$$

40b $$\begin{aligned}&\frac{\partial }{\partial z}\left( 3\mu A\frac{\partial v}{\partial z}\right) +\rho gA=0, \end{aligned}$$

40c $$\begin{aligned}&\frac{\partial }{\partial t}(R^{2})+\frac{\partial }{\partial z}(vR^{2})=\frac{p_{s}}{16\pi \mu A}\left( 16\pi ^{2}R^{4}-A^{2}\right) , \end{aligned}$$

with the initial conditions

40d $$\begin{aligned} A(z,t=0)=A_{0},\ R(z,t=0)=R_{0},\ \text {for all}\ z\in {[0,L]}, \end{aligned}$$

and the boundary conditions

40e $$\begin{aligned} A(z=0,t)=A_{0},\ R(z=0,t)=R_{0},\ v(z=0,t)=v_{0},\ v(z=L,t)=v_{L},\ \forall \ t\ge 0, \end{aligned}$$

where *L* is the length of the hot-forming zone, $p_{s}$ is the inside pressure, $\mu$ is the input viscosity, $v_{0}$ is the feeding and $v_{_{L}}$ is the drawing speed.

The system (40) gives us the velocity *v*, the cross-sectional area *A* and the average radius *R* of the glass tube. The width, denoted by *W*, of the glass tube can be determined by the relation

$$\begin{aligned} A=2\pi RW. \end{aligned}$$

Throughout the forming zone of the tubing process, the temperature remains constant and hence the viscosity $\mu$ of the molten glass is also remains constant.

### Appendix B: A motivating example

In order to understand our approach introduced, there arise two cases.

#### Case-I: when the exact solution is possible with the given BCs

Consider the following BVP

41 $$\begin{aligned} 2y'y''=1,\; y(0)=2,\; y(3)=\frac{20}{3}. \end{aligned}$$

It is possible to find the exact solution of Eq. (41) with the given boundary conditions, and is computed to give

42 $$\begin{aligned} y(x)=\frac{2}{3}(x+1)^{3/2}+\frac{4}{3}. \end{aligned}$$

We employ shooting method to convert the BVP (41) into an IVP

43 $$\begin{aligned} 2y'y''=1,\; y(0)=0,\; y'(0)=w_{0}\approx 1, \end{aligned}$$

where $w_{0}$ is approximated value determined by shooting the value of *y* at 3 i.e., $y(3)=\frac{20}{3}$. The developed analytical solution of IVP (43) is given as

44 $$\begin{aligned} y(x)=\frac{2}{3}(x+w_{0}^{2})^{3/2}+\frac{2}{3}(3-w_{0}^{3}), \end{aligned}$$

which is also a semi-analytic solution of the problem (41) using the proposed semi-analytic technique. The computed analytical solution (44) is then compared with both the numerical and exact solutions to better understand the accuracy of obtained solution as shown in Fig. 6. The corresponding errors are illustrated in Figs. 7 and 8.

#### Case-II: when the exact solution is impossible with the given BCs

In this case, it is not always possible to determine an analytical solution of a system of equations with the given initial and boundary conditions. As an example, we refer the reader to^22^.
